# Deep learning models based on CT predict prognosis in patients with locally advanced esophageal squamous cell carcinoma who received neoadjuvant immunotherapy and chemotherapy

**DOI:** 10.3389/fimmu.2026.1900089

**Published:** 2026-08-04

**Authors:** Junfeng Zhao, Zongqian Yuan, Huan Shi, Ying Li, Chunhui Li, Ruidan Zhang, Chengxin Liu

**Affiliations:** 1 Department of Radiation Oncology, Shandong Cancer Hospital and Institute, Shandong First Medical University and Shandong Academy of Medical Sciences, Jinan, Shandong, China; 2 Department of Oncology, Cancer Center, West China Hospital, Sichuan University, Chengdu, Sichuan, China; 3 Department of Gastroenterology, Shandong Cancer Hospital and Institute, Shandong First Medical University, Shandong Academy of Medical Science, Jinan, Shandong, China; 4 Department of Respiratory Oncology, Shandong Cancer Hospital and Institute, Shandong First Medical University and Shandong Academy of Medical Sciences, Jinan, Shandong, China; 5 Division of Thoracic Tumor Multimodality Treatment, Cancer Center, West China Hospital, Sichuan University, Chengdu, Sichuan, China

**Keywords:** esophageal squamous cell carcinoma, neoadjuvant, radiotherapy, ResNet-18, VISTA3D

## Abstract

**Objective:**

Neoadjuvant immunotherapy and chemotherapy (NICT) has emerged as the standard pharmacological intervention for locally advanced esophageal squamous cell carcinoma (ESCC). However, individual responses to this drug combination vary significantly. This study aims to develop a deep learning (DL) framework based on spatial radiomics to non-invasively profile tumor responses to NICT, thereby serving as a companion diagnostic to design optimal personalized combinatorial regimens.

**Methods:**

This retrospective study included 655 patients with locally advanced ESCC receiving NICT across two centers (January 2022–December 2024), divided into training, internal, and external validation cohorts. We utilized VISTA3D combined with point-prompt technology for precise primary tumor segmentation. Unsupervised clustering was then applied to delineate intratumoral heterogeneous “habitats”, which capture the microenvironmental phenotypes associated with immunochemotherapeutic response. A DL model based on ResNet18 and an attention-based multi-instance learning (MIL) framework was constructed to extract spatial radiomics features, predict disease-free survival (DFS), and stratify patients to guide the design of adjuvant therapeutic strategies.

**Results:**

The AI-assisted segmentation achieved a robust Dice similarity coefficient of 0.87 ± 0.18 in the external validation cohort. The ResNet18-based DL model demonstrated excellent prognostic performance (External C-index:0.85, 95% CI:0.71–0.95). By translating imaging phenotypes into risk profiles, the model successfully stratified patients: the low-risk group exhibited significantly superior DFS and OS compared to the high-risk group (P<0.001). Crucially, the model facilitated precise treatment regimen design. High-risk patients derived substantial survival benefits from the addition of adjuvant radiotherapy (DFS HR:0.52; OS HR:0.82), whereas low-risk patients showed no significant benefit, suggesting they could be spared from radiation-induced toxicities. Grad-CAM and SHAP analyses confirmed that the model’s predictions correlate with tumor invasion and microenvironmental heterogeneity.

**Conclusion:**

The proposed AI-driven spatial radiomics framework successfully bridges macroscopic imaging phenotypes with pharmacological responses to NICT. As an advanced companion diagnostic tool, it optimizes the design of multi-modality combinatorial regimens, preventing overtreatment while maximizing survival benefits for high-risk ESCC patients in the era of precision oncology.

## 1 Introduction

Esophageal cancer ranks as the ninth most common cancer globally and the sixth leading cause of cancer-related deaths. Over 50% of esophageal cancer patients are distributed in East Asia, with 90% of these cases being esophageal squamous cell carcinoma (ESCC). Surgery is widely recognized as the primary treatment for locally advanced resectable ESCC (1, 2). However, the prognosis for patients following surgery alone remains poor, with a five-year survival rate of only 25% (3–5). Based on the CROSS and NEOCRTEC5010 studies, the standard treatment regimen for locally advanced ESCC is now neoadjuvant chemoradiotherapy (NCRT) (6, 7). Although this approach significantly improves prognosis, it is still associated with a high recurrence rate and adverse effects such as anastomotic leakage (8). With the advent of the immunotherapy era, more options have emerged for neoadjuvant treatment regimens. Meta-analyses indicate that neoadjuvant immunotherapy and chemotherapy (NICT) demonstrate comparable efficacy to NCRT, while exhibiting a lower incidence of adverse events (9). Results from the ESCORT-NEO/NCCES01 trial demonstrated that the combination of camrelizumab with chemotherapy achieved a pathological complete response (pCR) rate of 28.0%, significantly higher than that in the neoadjuvant chemotherapy group, with manageable safety profiles (10). A retrospective analysis demonstrated that NICT is comparable to NCRT in terms of pathological response rate and R0 resection rate. Although NCRT achieves a more significant reduction in primary tumor staging, NICT exhibits superior three-year disease-free survival (DFS) and overall survival (OS), indicating better long-term prognosis (11). These findings suggest that NICT may confer more significant survival benefits to patients. In parallel with these clinical advances, a growing body of research has investigated the utility of radiomics and deep learning (DL) for predicting treatment response in ESCC patients receiving neoadjuvant immunochemotherapy (12–15). Several multicenter studies have reported promising performance in predicting pCR following NICT, employing various DL architectures including Vision-Mamba-based models, habitat-aware radiomics with adaptive 2.5D DL, delta-radiomics combined with inflammatory biomarkers, and sub-regional radiomics integrated with multichannel 2D/3D DL (12–15). These models have achieved AUCs ranging from 0.79 to 0.92 across independent validation cohorts, demonstrating the feasibility of non-invasive response prediction. Despite these encouraging advances, several critical gaps persist. First, the vast majority of existing models focus on predicting pCR as the primary endpoint, whereas pCR—although associated with improved survival—has not been conclusively validated as a surrogate for long-term prognosis (16). Few studies have directly modeled DFS or OS as the primary outcome in the NICT setting. Second, no published study has employed a DL-based prognostic model to guide postoperative adjuvant radiotherapy selection in ESCC patients receiving NICT, representing a significant unmet clinical need given the ongoing debate regarding adjuvant therapy indications. Third, most radiomics studies rely on manual or semi-automated tumor segmentation, which substantially limits clinical scalability and real-world implementation. The integration of fully automated segmentation pipelines with prognosis prediction remains largely unexplored. Therefore, the ability to predict patient prognosis prior to NICT treatment could provide valuable guidance for clinical decision-making.

Beyond the comparative efficacy between NCRT and NICT, the immunological underpinnings of ESCC response to neoadjuvant immunotherapy warrant deeper consideration. ESCC is characterized by substantial intratumoral heterogeneity (ITH), encompassing genetic, phenotypic, and spatial diversity that profoundly influences the tumor immune microenvironment (TIME). Emerging evidence indicates that the efficacy of immune checkpoint inhibitors (ICIs) in ESCC is intricately modulated by the baseline immune landscape, including PD-L1 combined positive score (CPS), the density and spatial distribution of tumor-infiltrating lymphocytes (TILs), and the presence of tertiary lymphoid structures (TLS) (17). Notably, NICT exerts dual effects: cytotoxic chemotherapy induces immunogenic cell death (ICD), releasing tumor-associated antigens and damage-associated molecular patterns (DAMPs) that prime T-cell responses, while ICIs relieve adaptive immune resistance, synergistically reshaping the TIME (18). However, this dynamic immunological remodeling is highly heterogeneous across individuals, leading to divergent therapeutic outcomes. Spatial heterogeneity within the tumor—manifested as distinct subregions (or ‘habitats’) with varying degrees of immune infiltration, hypoxia, necrosis, and stromal composition—may dictate regional variations in ICI sensitivity and resistance mechanisms (19). Conventional biopsy-based assessments capture only focal snapshots and fail to represent this global spatial heterogeneity. Therefore, non-invasive imaging-based approaches that delineate intratumoral habitat heterogeneity offer a promising avenue to holistically profile the TIME and predict immunotherapeutic responses. This rationale underpins our use of CT-based radiomic habitat analysis to non-invasively decode microenvironmental phenotypes associated with NICT responsiveness in locally advanced ESCC.

Although neoadjuvant therapy has demonstrated significant benefits for patients with locally advanced ESCC, the role of postoperative adjuvant therapy remains under investigation. A real-world study indicates that ESCC patients who did not achieve pathological complete response (non-pCR) after NCRT experienced significant improvements in OS and DFS when receiving postoperative adjuvant chemotherapy (20). However, whether patients who have already received NICT can benefit from subsequent adjuvant therapy remains controversial.

Although the NEOCRTEC5O10 study demonstrated improved patient survival rates, 32% to 49% of patients still face a risk of recurrence (21). This indicates that ESCC patients receiving NCRT still require postoperative adjuvant therapy. The CheckMate-577 study provides a significant complement to the CROSS treatment model, substantially improving patient survival benefits (22). For patients with ESCC who have received surgical treatment, postoperative adjuvant radiotherapy is primarily recommended for high-risk cases, such as those with postoperative staging of T3–4 or R1 resection. Currently, there are no large-scale clinical trials guiding the application of adjuvant radiotherapy following NICT. To address these identified gaps, this study proposes a two-stage automated prediction framework for DFS based on ‘image segmentation—prognosis prediction’. Unlike prior studies that predominantly focus on pCR prediction, we directly model DFS as the primary prognostic endpoint and, for the first time, demonstrate that DL-based risk stratification can identify patients who derive significant survival benefits from postoperative adjuvant radiotherapy, thereby addressing the critical clinical question of adjuvant therapy selection following NICT. Additionally, we integrate VISTA3D automated segmentation with point-prompt technology to overcome the manual segmentation bottleneck, achieving robust Dice coefficients and enabling clinical scalability. The study confirmed that combining point-prompt segmentation with an automated segmentation model, integrated with a CT-based ResNet18 prediction model, effectively predicts DFS in ESCC patients receiving NICT and efficiently identifies patients likely to benefit from adjuvant radiotherapy, offering a precision oncology approach to postoperative management.

## 2 Methods and materials

### 2.1 Patients

**Figure 1:**
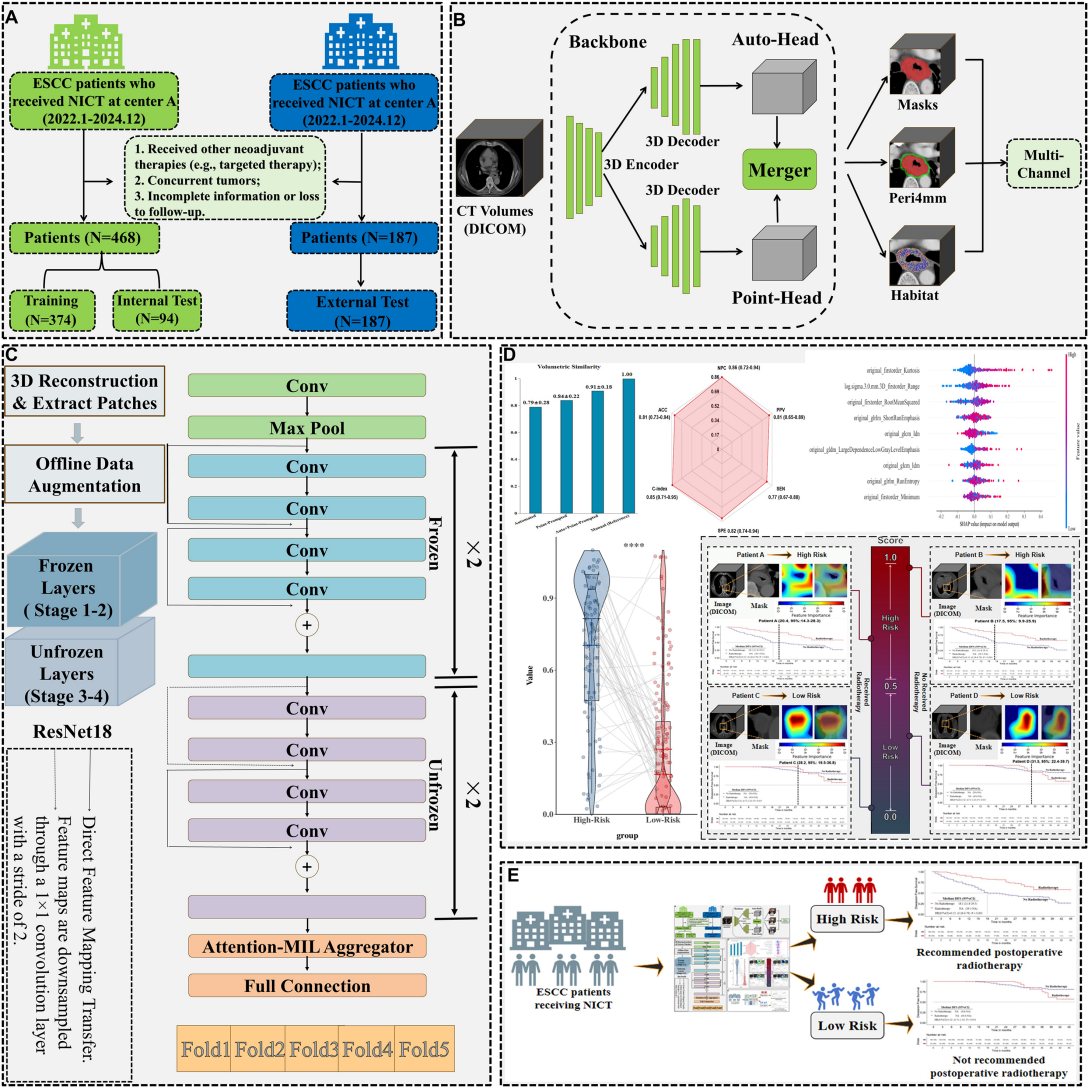
Overview of the study design and analytical workflow. **(A)** Patient inclusion. **(B)** Segmentation model utilizing the VISTA3D model. **(C)** Deep learning model based on ResNet18 feature extraction and an attention-based multi-instance learning framework. **(D)** Evaluation of model performance. **(E)** Development of a decision pathway based on this study.

This study retrospectively analyzed patients with locally advanced ESCC who received NICT from January 2022 to December 2024 at two centers: Center A (468 patients) and Center B (187 patients). Inclusion criteria were: age ≥18 years; pathologically confirmed ESCC; receipt of NICT; and availability of pre-treatment CT imaging. Patients were excluded if they had unresectable tumors or metastases during exploratory surgery, received other neoadjuvant therapies (e.g., targeted therapy), had concurrent tumors, or had incomplete information or were lost to follow-up. Patients from Center A were divided into a training cohort and an internal validation cohort in an 8:2 ratio using stratified random sampling. Stratification was performed based on key prognostic factors, including age group (<60 vs. ≥60 years), sex, clinical T stage, clinical N stage, and pCR status, to ensure balanced distribution of these covariates across the two cohorts. The stratified split was implemented using the stratified shuffle split function from the scikit-learn library (version 1.2.1) with a fixed random seed (42) for reproducibility. Center B served as the external validation cohort, with all patients included without any sample selection or stratification to preserve its natural distribution for assessing model generalizability. As shown in **Table 1**, no significant differences were observed across the training, internal validation, and external validation cohorts for all clinical and pathological variables (all P > 0.05), confirming baseline balance among cohorts. All patients received postoperative adjuvant systemic therapy and were divided into radiotherapy and no-radiotherapy groups based on whether they received postoperative adjuvant radiotherapy. **Figure 1** presents the design framework for this study. Detailed treatment protocols for all patients are provided in **Supplementary Appendix 1**. The work has been reported in line with the STROCSS criteria (23).

**Table 1:** Baseline characteristic information.

Variables	Total (n = 655)	Training (n = 374)	Internal test (n = 94)	External test (n = 187)	*P*
Age, n(%)					0.051
<60	211 (32.21)	107 (28.61)	38 (40.43)	66 (35.29)	
≥60	444 (67.79)	267 (71.39)	56 (59.57)	121 (64.71)	
Sex, n(%)					0.067
Female	105 (16.03)	56 (14.97)	18 (19.15)	31 (16.58)	
Male	550 (83.97)	318 (85.03)	76 (80.85)	156 (83.42)	
Smoking, n (%)					0.383
No	296 (45.19)	161 (43.05)	43 (45.74)	92 (49.20)	
Yes	359 (54.81)	213 (56.95)	51 (54.26)	95 (50.80)	
KPS, n (%)					0.211
<90	443 (67.63)	245 (65.51)	62 (65.96)	136 (72.73)	
≥90	212 (32.37)	129 (34.49)	32 (34.04)	51 (27.27)	
T, n(%)					0.124
1	7 (1.07)	7 (1.87)	0 (0.00)	0 (0.00)	
2	45 (6.87)	22 (5.88)	5 (5.32)	18 (9.63)	
3	569 (86.87)	328 (87.70)	82 (87.23)	159 (85.02)	
4	34 (5.19)	17 (4.55)	7 (7.45)	10 (5.35)	
N, n(%)					0.686
0	109 (16.64)	68 (18.18)	15 (15.96)	26 (13.90)	
1	234 (35.73)	131 (35.03)	31 (32.98)	72 (38.50)	
2	298 (45.50)	166 (44.39)	45 (47.87)	87 (46.52)	
3	14 (2.14)	9 (2.41)	3 (3.19)	2 (1.07)	
Location, n(%)					0.466
Lower	356 (54.35)	198 (52.94)	56 (59.57)	102 (54.55)	
Middle	270 (41.22)	157 (41.98)	37 (39.36)	76 (40.64)	
Upper	29 (4.43)	19 (5.08)	1 (1.06)	9 (4.81)	
Surgery, n(%)					0.119
Minimally invasive surgery	549 (83.82)	304 (81.28)	83 (88.30)	162 (86.63)	
Thoracotomy	106 (16.18)	70 (18.72)	11 (11.70)	25 (13.37)	
Perineural Invasion, n(%)					0.309
No	559 (85.34)	319 (85.29)	76 (80.85)	164 (87.70)	
Yes	96 (14.66)	55 (14.71)	18 (19.15)	23 (12.30)	
Lymphovascular Invasion, n(%)					0.289
No	512 (78.17)	300 (80.21)	69 (73.40)	143 (76.47)	
Yes	143 (21.83)	74 (19.79)	25 (26.60)	44 (23.53)	
PCR, n(%)					0.711
No	489 (74.66)	282 (75.40)	67 (71.28)	140 (74.87)	
Yes	166 (25.34)	92 (24.60)	27 (28.72)	47 (25.13)	
Neoadjuvant treatment cycle, n(%)					0.953
>2	127 (19.39)	74 (19.79)	18 (19.15)	35 (18.72)	
≤2	528 (80.61)	300 (80.21)	76 (80.85)	152 (81.28)	
Tumor length, Mean ± SD	6.03 ± 2.40	5.98 ± 2.47	5.86 ± 2.33	6.21 ± 2.30	0.432

### 2.2 Follow-up and endpoint definitions

All patients were regularly followed up after surgery according to a standardized protocol. Follow-up evaluations were conducted every 3 months during the first 2 years post-surgery, every 6 months during years 3 to 5, and annually thereafter. Each follow-up visit included physical examination, routine blood tests, tumor marker assays, and contrast-enhanced CT of the chest and upper abdomen. Additional examinations-including esophagoscopy, endoscopic ultrasonography, or positron emission tomography-CT were performed when clinically indicated to confirm suspected recurrence or metastasis. All follow-up data were prospectively collected into the institutional database by dedicated research nurses, and survival status was further verified through the national death registry system when necessary.

The primary endpoint of this study was DFS, defined as the time from the date of surgery to the date of the first documented disease recurrence (locoregional or distant), secondary malignancy, or death from any cause, whichever occurred first. The secondary endpoint was OS, defined as the time from the date of surgery to the date of death from any cause. Patients who were alive and free of disease at the last follow-up were censored at the date of their last contact. Patients lost to follow-up were censored at the date of their last known contact. The follow-up cut-off date was December 31, 2025. At the time of data lock, the median follow-up duration for censored patients was 31.4 months.

### 2.3 CT image acquisition and preprocessing

All patients underwent CT imaging prior to receiving neoadjuvant therapy. The acquired CT images were uploaded to the Picture Archiving and Communication System (PACS) and exported in DICOM format. Where multiple CT scans existed, the scan closest to the treatment date was used. All CT images underwent standardized pre-processing to ensure data consistency. To mitigate the impact of variations in voxel spacing, spatial normalization is commonly employed. To address such issues, this study applied fixed-resolution resampling techniques. All images were resampled to a voxel size of 1 mm × 1 mm × 1 mm to achieve spacing normalization. Two senior radiation oncologists with ten years of experience manually delineated the primary tumor regions on CT images using ITK-SNAP 4.4.0 software (https://www.itksnap.org/). To evaluate inter-observer delineation consistency, a random subset of 50 cases (approximately 7.6% of the total cohort) was independently annotated by both physicians, who were blinded to each other’s delineations and to patient clinical outcomes. Inter-observer agreement was assessed using the Dice Similarity Coefficient for spatial overlap. The mean Dice Coefficient was 0.91 (standard deviation: 0.15), indicating excellent inter-observer agreement. All cases with a DSC below 0.80 or volume discrepancy exceeding 10% were jointly re-evaluated by both physicians along with a third senior radiologist to reach a consensus annotation. The consensus annotations were used as the reference standard for all subsequent segmentation evaluation and model development.

### 2.4 VISTA3D segmentation deep learning model

**Figure 2:**
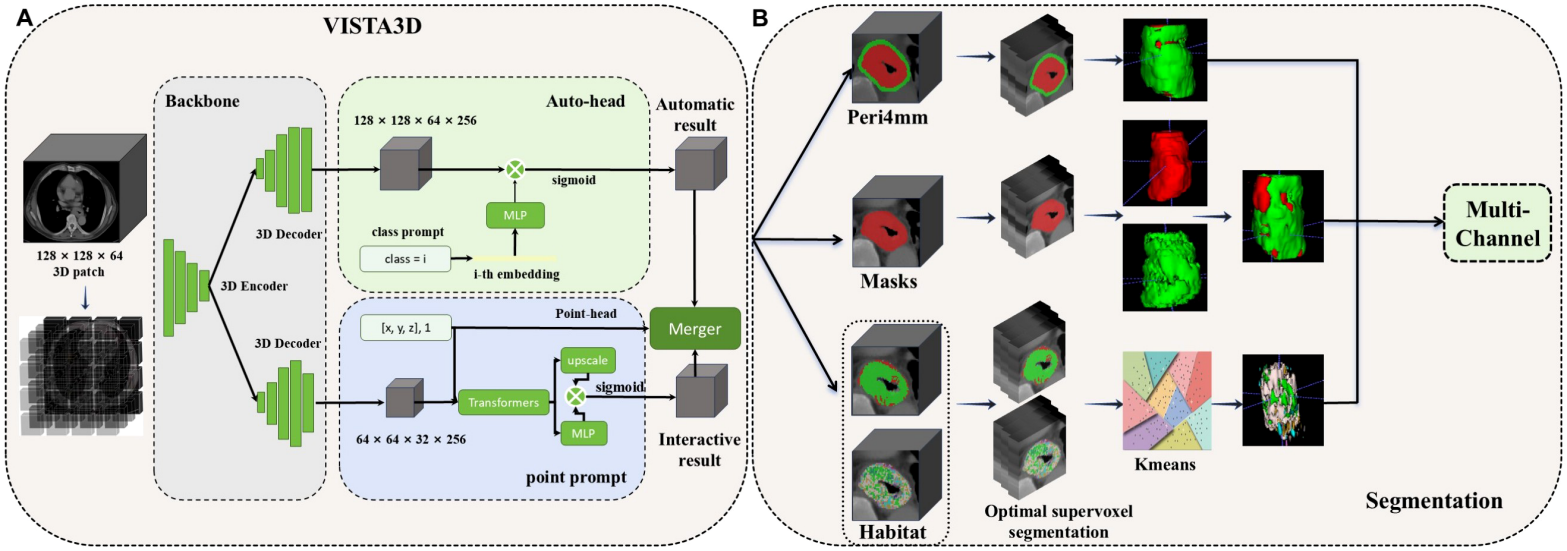
Automatic segmentation and habitat analysis. **(A) **VISTA3D Architecture. It comprises two branches sharing the same image encoder. If the user provides a class prompt within the 127 supported classes, the top automatic branch activates pre-trained automatic segmentation. If the user provides a 3D point prompt, the bottom interactive branch activates interactive segmentation. If both branches are activated, the fusion module uses the interactive results to edit the automatic results. **(B)** The VISTA3D-based automated segmentation architecture not only segments the tumor lesion itself but also constructs constructed a 3mm peritumoral ring, dividing both the tumor and peritumoral ring into multiple subregions. The K-means clustering algorithm is employed to partition all voxels within the tumor into K distinct clusters.

This study employs VISTA3D, a unified segmentation framework for 3D medical images based on the SegResNet backbone and sliding-window inference (**Figure 2A**). Through the integration of 3D super-voxel distillation knowledge and a four-stage training process, VISTA3D achieves the automatic segmentation of 127 structural classes, 3D interactive refinement and zero-shot segmentation. The reliability analysis in this study focuses on the results of the three core segmentation methods supported by VISTA3D, which are compared against manually annotated gold standards on an external test dataset. The specific sources are as follows: Firstly, automatic segmentation leverages the shared SegResNet backbone and auto-branching to perform end-to-end segmentation of target structures without manual intervention. Secondly, point-prompt segmentation (i.e. interactive segmentation). Thirdly, automatic segmentation combined with point-prompt segmentation. This study selected four widely recognized quantitative metrics in medical image segmentation: The Dice Similarity Coefficient, the 95% Hausdorff Distance, the Mean Surface Distance and the Volume Similarity. These metrics were used to calculate consistency between the three segmentation results and manual segmentation. Detailed protocols are provided in **Supplementary Appendix 2**.

### 2.5 Automated tumor habitat segmentation on CT

In this study, we constructed a 3 mm peritumoral ring based on the primary lesion, subdividing the tumour and peritumoral ring into multiple subregions. Specifically, we first obtained the binary mask of the primary tumor from the segmentation results. To generate the 3 mm peritumoral ring, we applied a dilation operation using a spherical structuring element with a radius of 3 mm (equivalent to 3 voxels, as all images were resampled to 1 mm × 1 mm × 1 mm isotropic resolution). The dilated mask was then subjected to a hole-filling operation to ensure a complete and contiguous annular region. Subsequently, we applied a subtraction operation to remove the original tumor mask from the dilated mask, yielding the peritumoral annular region with a thickness of exactly 3 mm. This region was then combined with the original tumor mask to create the final habitat segmentation volume. **Figure 2B** illustrates the resulting expanded peritumoral region and its subregions. To capture intratumoral heterogeneity, we used unsupervised clustering algorithms to divide voxels within each tumour into groups. First, we used principal component analysis to reduce the dimensionality of the high-dimensional features, retaining components that explained 95% of the total variance, thereby eliminating redundancy and accelerating computation. Next, the K-means clustering algorithm was used to partition all the voxels within the tumour into K different clusters. Each cluster represents a ‘habitat’ with a unique radiological phenotype.

To determine the optimal number of clusters K, we employed a combined approach using the elbow method and the silhouette coefficient. Specifically, we evaluated K values ranging from 2 to 30, covering a broad spectrum from biologically minimal to potentially over-segmented habitat partitions. For each K value, we computed the within-cluster sum of squares (WCSS) for the elbow method and the average silhouette coefficient across all samples. The optimal K was selected at the elbow point where the marginal decrease in WCSS substantially diminished, in conjunction with the K value yielding the highest average silhouette coefficient. In our dataset, the elbow curve exhibited a clear inflection point at K = 3, with the rate of WCSS decrease diminishing markedly beyond this value. The silhouette coefficient reached its maximum at K = 3 (mean silhouette score: 0.42), compared to 0.38 for K = 2, 0.39 for K = 4, and progressively lower values for larger K (ranging from 0.31 to 0.27 for K = 5 to K = 30). Based on these combined criteria, K = 3 was selected as the optimal number of habitats.

To further validate that the chosen K value was optimal for the downstream prognostic task, we performed a sensitivity analysis by constructing separate prognostic models using habitat segmentations derived from representative K values (K = 2, 3, 4, 5, 10, and 20). The model performance, evaluated by the C-index in the internal validation cohort, was 0.83 for K = 2, 0.87 for K = 3, 0.85 for K = 4, 0.84 for K = 5, 0.81 for K = 10, and 0.79 for K = 20, indicating that K = 3 achieved the best prognostic performance. This sensitivity analysis confirmed that the K value selected by the elbow and silhouette criteria also yielded optimal performance in the downstream DL prognostic model, while larger K values introduced over-segmentation and degraded predictive performance.

### 2.6 Multiple instance learning based on ImageNet

The pydicom library was used to read DICOM slice sequences from each patient’s folder. All slices were numerically sorted to accurately reconstruct anatomically continuous 3D CT volumes. This study employs ResNet18 (an 18-layer residual network pre-trained on ImageNet) as the feature extraction backbone (24). To balance the retention of pre-trained knowledge with adaptation to CT image features, a hierarchical freezing strategy is adopted. In the final configuration, the shallow-stage layers of the model are frozen, while only the two deeper-stage layers are unfrozen to participate in gradient updates. We implemented an aggregator based on AttentionMIL. This module consists of a two-layer Multi-Layer Perceptron (MLP) and a Softmax activation function.

This study employs the Cox Proportional Hazards Loss Function (CoxPHLoss) to evaluate the predictive capability of the model (25). This study employed 5-fold cross-validation to evaluate model performance. During data fold partitioning, stratification was performed based on the “death” event indicator to ensure that the ratio of events to censored cases in each fold remained consistent with the overall dataset. The primary evaluation metric was Harrell’s C-index, which quantifies the concordance between the model’s predicted risk rankings and the actual observed survival time rankings (**Figure 1C**). The study also evaluated the model’s predictive performance using Negative Predictive Value (NPV), Positive Predictive Value (PPV), Accuracy (ACC), Sensitivity (SEN), Specificity (SPE), and calibration curves. Detailed protocols are provided in **Supplementary Appendix 3**.

To deeply understand the basis for model decisions and enhance its clinical credibility, this study employs a combined approach of Gradient-Weighted Class Activation Maps (Grad-CAM) and SHapley Additive Explanations (SHAP). This approach explains the model’s predictive behavior from two dimensions: spatial regions within the image and feature importance. Grad-CAM is employed to visualize spatial regions within input CT images that significantly contribute to the model’s prognostic risk prediction. Specifically, based on the trained ResNet18-MIL model, we compute the gradient information of the final convolutional layer’s activation map and generate a heatmap through weighted aggregation. This heatmap visually highlights the anatomical regions (such as tumor core, infiltrative margins, surrounding stroma, or lymph node areas) that the model prioritizes when predicting a patient’s prognostic risk following NICT. By comparing the heatmap distributions across different risk-stratified patient groups, clinicians can visually discern spatial patterns of focus in the model’s prognostic assessment. This enables them to infer potential imaging-based biological features associated with the tumor immune microenvironment or treatment response. To further uncover the key radiomic features influencing model predictions, this study employs SHAP analysis to assess feature importance holistically. Based on radiomic features extracted during training, we calculate the SHAP values for each feature’s contribution to model outputs, thereby quantifying their individual contributions (**Supplementary Appendix 3**).

### 2.7 Identify patients who may benefit from adjuvant radiotherapy

The model ultimately outputs a deep learning (DL) score for each individual. By combining this score with the patient’s DFS, a cutoff value is derived. Based on this cutoff, patients are categorized into low-risk (favorable prognosis) and high-risk (poor prognosis) groups. Kaplan-Meier survival analysis is then employed to evaluate the prognosis of both groups, followed by subgroup analysis. Kaplan-Meier survival analyses were conducted in low-risk and high-risk patients, respectively, to evaluate the benefit of adjuvant radiotherapy according to whether the patients received adjuvant radiotherapy. Finally, a clinical decision pathway for patients with locally advanced ESCC undergoing NICT was established in this study.

### 2.8 Statistical analysis

For statistical analysis, the Python statsmodels (version 3.7.12) module was utilized, and a P value < 0.05 was deemed statistically significant. We analyzed the differences between groups using the Student’s t-test or Mann−Whitney U test for continuous variables and the chi-square test or Fisher’s exact test for categorical variables. Kaplan–Meier analysis was used to evaluate DFS and OS.

## 3 Results

### 3.1 Patient characteristics

The study ultimately included 655 patients, comprising 374 in the training cohort, 94 in the internal validation cohort, and 187 in the external validation cohort. By the end of follow-up, 273 (41.68%) patients experienced disease progression, and 157 (23.97%) patients experienced death. Among the three patient cohorts, 267 (71.39%), 56 (59.57%), and 121 (64.71%) patients were aged ≥60 years, respectively. Male patients constituted the majority in all three groups: 318 (85.03%), 76 (80.85%), and 156 (83.42%) patients, respectively; Postoperative pathological complete response (pCR) was achieved in 92 (24.60%), 27 (28.72%), and 47 (25.13%) patients, respectively (**Table 1**).

### 3.2 Performance of the 3D automated segmentation

**Figure 3:**
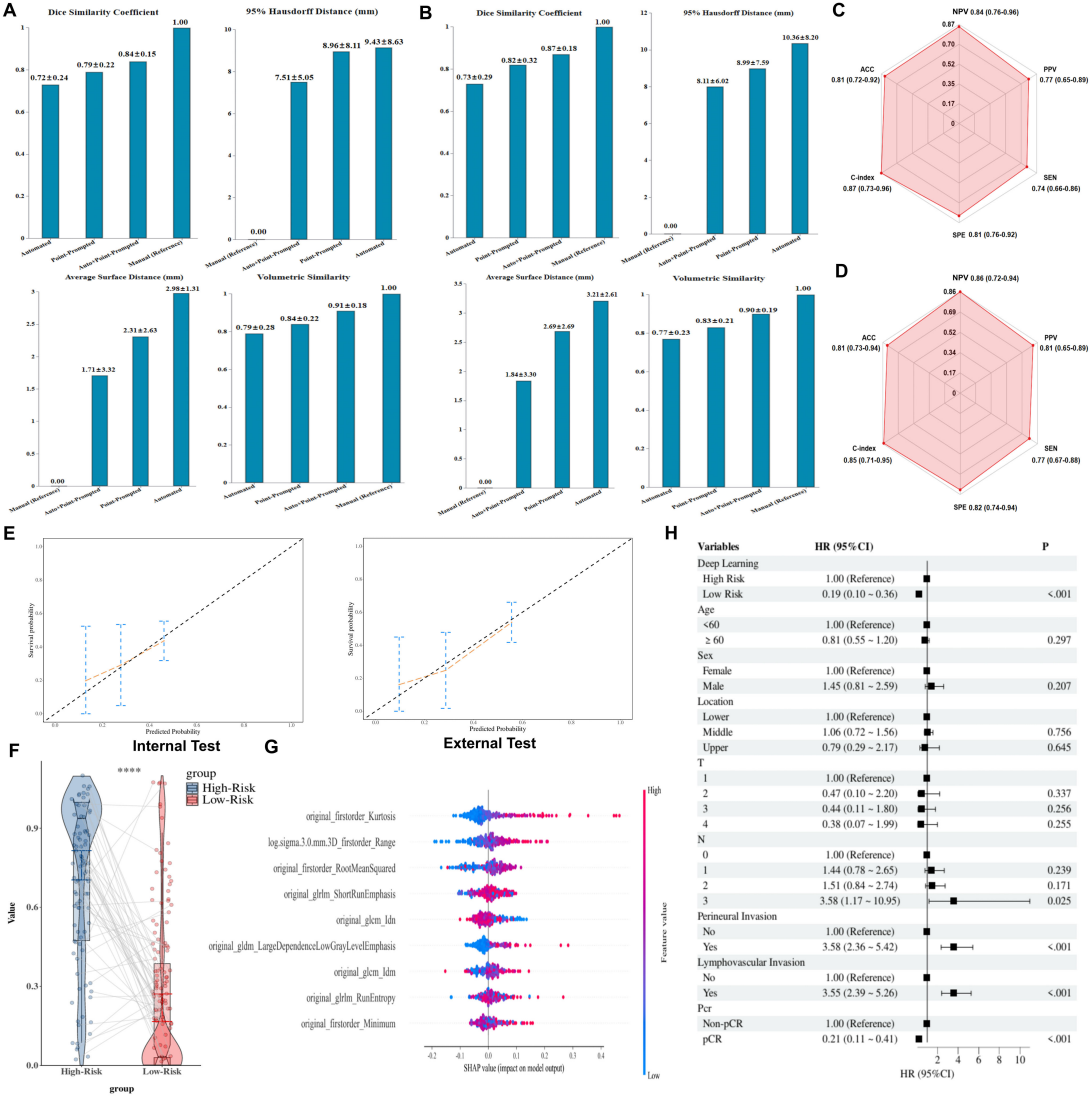
Evaluation of model performance. **(A)** Comparison of segmentation performance among three segmentation models in the internal validation cohort. **(B)** Comparison of segmentation performance among three segmentation models in the external validation cohort. **(C)** Radar chart showing the predictive performance of deep learning models in the internal validation cohort. **(D)** Radar chart showing the predictive performance of deep learning models in the external validation cohort. **(E)** Calibration curve of model prediction performance. **(F)** Violin plot of deep learning score distribution for low-risk and high-risk patients. **(G)** SHapley Additive exPlanations for the selected features. **(H)** Forest plot of COX regression analysis.

The segmentation performance of three core segmentation methods was evaluated using internal and external validation cohorts. Comparative results demonstrate that the point-prompt combined with automated segmentation approach effectively resolves misclassification issues inherent in standalone automated segmentation while maintaining automated segmentation efficiency. Within the internal validation cohort, this method achieved a Dice similarity coefficient of 0.84 ± 0.15, representing a 12% and 5% improvement over independent automatic segmentation and point-prompt segmentation, respectively (**Figure 3A**). In the external validation cohort, this method achieved a Dice similarity coefficient of 0.87 ± 0.18, representing a 14% and 5% improvement over independent automatic segmentation and point-prompt, respectively (**Figure 3B**). The method achieves significant improvements in 95% Hausdorff distance, mean surface distance, and volume similarity metrics. Furthermore, this method substantially reduces manual interaction costs compared to independent point-prompt segmentation, making it the chosen implementation for subsequent automated segmentation-based DFS prediction tasks.

### 3.3 Model performance

In the internal validation cohort, the DL model achieved a C-index of 0.87 (95% confidence interval [CI]: 0.73–0.96), an ACC of 0.81 (95% CI: 0.72–0.92), an NPV of 0.84 (95% CI: 0.76–0.96), PPV was 0.77 (95% CI: 0.65–0.89), SEN was 0.74 (95% CI: 0.66–0.86), and SPE was 0.81 (95% CI: 0.76–0.92) (**Figure 3C**). In the external validation cohort, the DL model achieved a C-index of 0.85 (95% CI: 0.71–0.95), an ACC of 0.81 (95% CI: 0.73–0.94), an NPV of 0.86 (95% CI: 0.72–0.94), PPV was 0.81 (95% CI: 0.65–0.89), SEN was 0.77 (95% CI: 0.67–0.88), and SPE was 0.82 (95% CI: 0.74–0.94) (**Figure 3D**). The calibration curve of the DL model exhibits a distinct trend (**Figure 3E**). This study generated DL model scores for all patients. Based on these scores and DFS outcomes across the entire cohort, a cutoff value of 0.53 was determined, dividing the total cohort into a high-risk group (452 cases) and a low-risk group (203 cases). Significant differences in scores were observed between the two groups (**Figure 3F**). By calculating the absolute mean SHAP values for each feature, we identified the radiomic features with the most significant impact on model output (**Figure 3G**). In the multivariate Cox regression analysis, the DL model emerged as an independent predictor of DFS (**Figure 3H**).

Prior to evaluating the model on the held-out internal and external validation cohorts, we performed 5-fold stratified cross-validation on the training cohort to assess model stability and generalizability. Across the five folds, the model achieved a mean C-index of 0.85 (standard deviation: 0.03, range: 0.81–0.89). The mean ACC was 0.79 (SD: 0.04), mean SEN was 0.72 (SD: 0.05), and mean SPE was 0.80 (SD: 0.04). The consistency of performance metrics across folds confirmed the stability of our model and the robustness of the training procedure. The complete fold-wise results are provided in **Supplementary Table 1**.

To reveal the visual attention patterns of the model when predicting patients with different prognostic risks, this study conducted a systematic analysis of Grad-CAM heatmaps for high-risk and low-risk groups. Results indicate that in Grad-CAM heatmaps of high-risk patients, the model’s areas of significant focus predominantly concentrate on the esophageal wall surrounding the tumor and adjacent mediastinal structures. High activation zones primarily distribute within 3–5 mm beyond the tumor margin, encompassing the entire thickness of the esophageal wall, surrounding adipose tissue, the adjacent tracheal membrane, the area adjacent to the aortic arch, and the mediastinal lymph node drainage regions. This spatial attention pattern suggests that the model may rely more heavily on imaging features such as tumor invasion extent, signs of surrounding tissue infiltration, and regional lymph node involvement when predicting poor prognosis in this patient group. This aligns with the clinical and pathological characteristics of ESCC, including local progression, lymph node metastasis, and poor response to immunotherapy. In contrast, among low-risk patients, the focus of Grad-CAM heatmaps is markedly concentrated on the internal structure of the tumor. High-intensity activation regions are predominantly confined to the tumor core. This indicates that the model tends to utilize tumor-internal heterogeneity features—such as the extent of internal necrosis, enhancement homogeneity, and depth of submucosal invasion—when identifying patients with favorable prognosis. These characteristics may reflect relatively localized tumor biology, active immune cell infiltration, or heightened sensitivity to neoadjuvant therapy. By comparing the heatmap distribution patterns between the two patient groups, this study further supports the model’s ability to provide anatomically and pathophysiologically relevant visual interpretations during prognostic stratification. Grad-CAM results indicate that tumor perivascular invasion serves as a critical imaging biomarker for high-risk patients, while tumor internal structural heterogeneity correlates with favorable prognosis. This provides visual evidence for future research into more targeted imaging prognostic markers for ESCC.

To quantitatively validate the spatial attention patterns observed in the Grad-CAM heatmaps, we performed a batch-level statistical analysis across all patients in the combined validation cohorts (internal + external, n = 281). High-risk patients (n = 189) exhibited predominantly peritumoral/distant-focused attention in 152 cases (80.4%), with the highest activation intensities concentrated in the peritumoral 3 mm ring (mean proportion of top-10% activation voxels: 52.3% ± 12.8%). In contrast, among low-risk patients (n = 92), 71 cases (77.2%) exhibited predominantly intratumoral-focused attention, with high activation voxels concentrated within the tumor core (mean proportion: 61.5% ± 14.2%). The difference in the proportion of peritumoral/distant-focused attention between high-risk and low-risk patients was statistically significant (80.4% vs. 22.8%, P < 0.001, chi-square test). These quantitative findings corroborate our qualitative observations and provide robust statistical evidence that the model’s spatial attention patterns systematically differ between prognostic risk groups.

**Figure 4:**
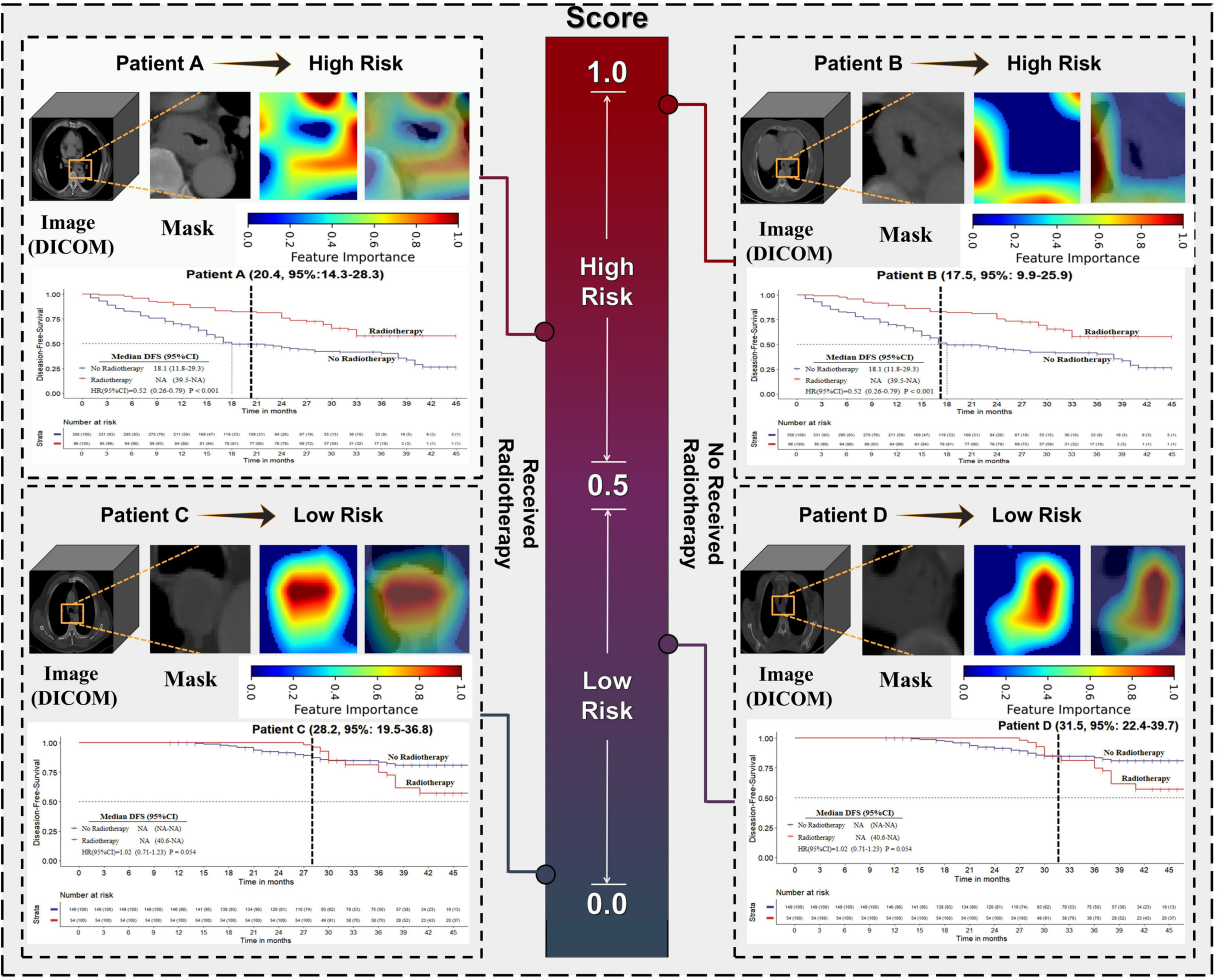
Model interpretability analysis. Patients A and B were classified as high-risk by the model; Patients C and D were classified as low-risk. The Gradient-weighted Class Activation Mapping visually represents the model's focus on lesions.

In the overall population survival analysis, DFS analysis demonstrated that low-risk patients had significantly better outcomes than high-risk patients (hazard ratio [HR]: 0.21, 95% CI: 0.15–0.30, P < 0.001). OS analysis revealed that low-risk patients also demonstrated significantly superior outcomes compared to high-risk patients (HR: 0.40, 95% CI: 0.27–0.59, P < 0.001). Forest plots from subgroup analyses showed consistent results across all subgroups, indicating a benefit for low-risk patients (**Figure 4**).

To directly evaluate whether segmentation errors from the automated pipeline propagate to and compromise downstream prognostic performance, we compared the predictive performance of the DL model using features derived from automated segmentation versus those derived from manual delineation in the external validation cohort. The manual segmentation-based model achieved a C-index of 0.86 (95% CI: 0.72–0.95), while the automated segmentation-based model achieved a C-index of 0.85 (95% CI: 0.71–0.95). DeLong’s test for correlated ROC curves revealed no statistically significant difference between the two models (P = 0.43), indicating that the automated segmentation pipeline provides prognostic information statistically equivalent to the expert manual gold standard. This finding empirically confirms that the inherent segmentation errors (Dice: 0.87 ± 0.18) do not substantially compromise the predictive capability of the downstream prognostic model.

### 3.4 Screening of patients who could benefit from radiotherapy

#### 3.4.1 High-risk patients

**Figure 5:**
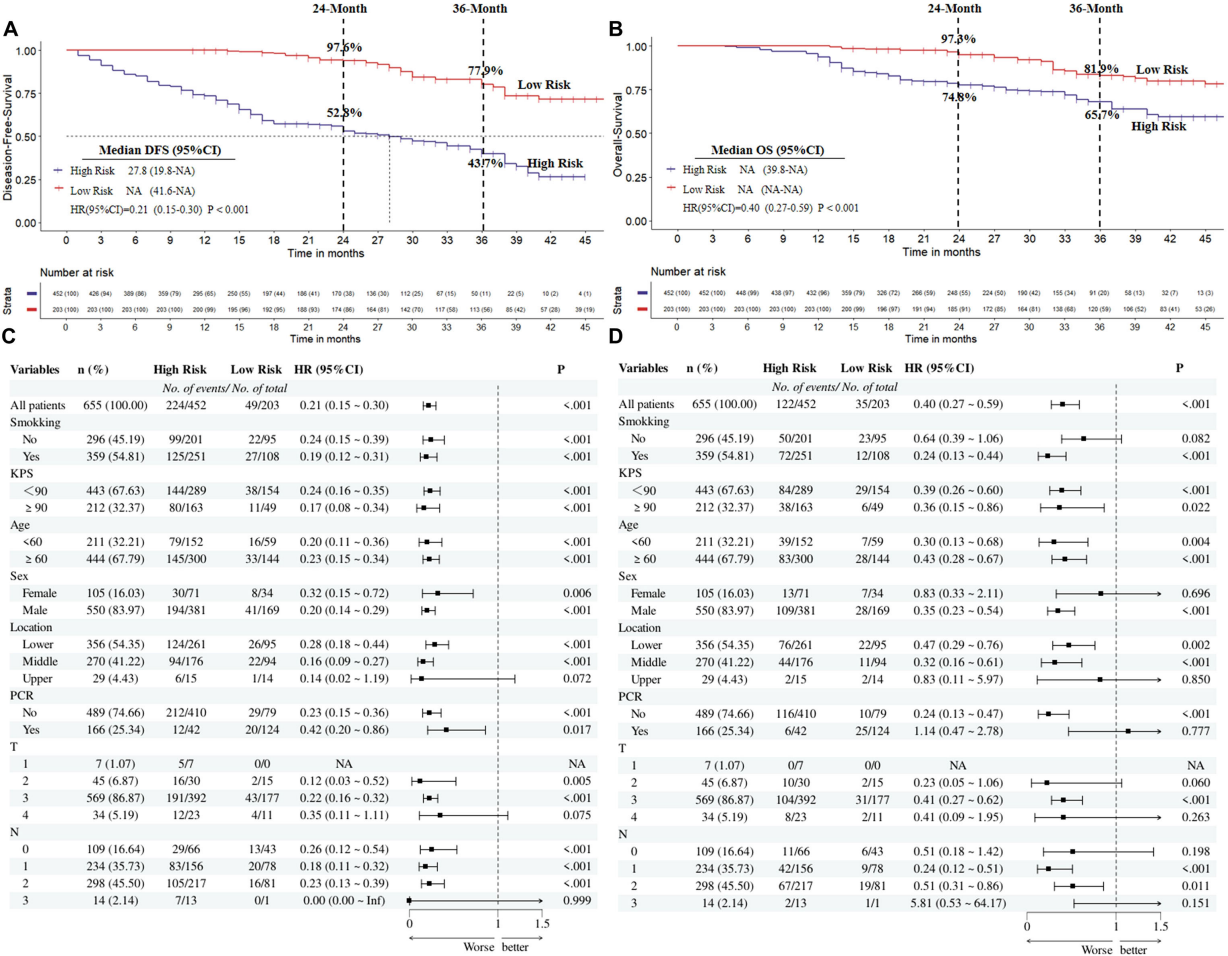
**(A)** Kaplan–Meier survival analysis of DFS in low-risk and high-risk cohorts. **(B)** Kaplan–Meier survival analysis of OS in low-risk and high-risk cohorts. **(C)** Subgroup analysis of DFS. **(D)** Subgroup analysis of OS.

Among the 452 patients assessed as high risk by the DL model, 96 (21.24%) received radiotherapy, while 356 (78.76%) did not. Analysis of DFS reveals that patients receiving radiotherapy demonstrated significantly superior median DFS compared to those not receiving radiotherapy (NA [95% CI: 39.5–NA] vs. 18.1 [95% CI: 11.8–29.3], HR: 0.52 [95% CI: 0.26–0.79], P < 0.001). Analysis of OS revealed similar findings (NA [95% CI: 42.6–NA] vs. 42.0 [95% CI: 31.6–NA], HR: 0.82 [95% CI: 0.63–0.98], P = 0.008) (**Figures 5A, B**).

#### 3.4.2 Low-risk patients

**Figure 6:**
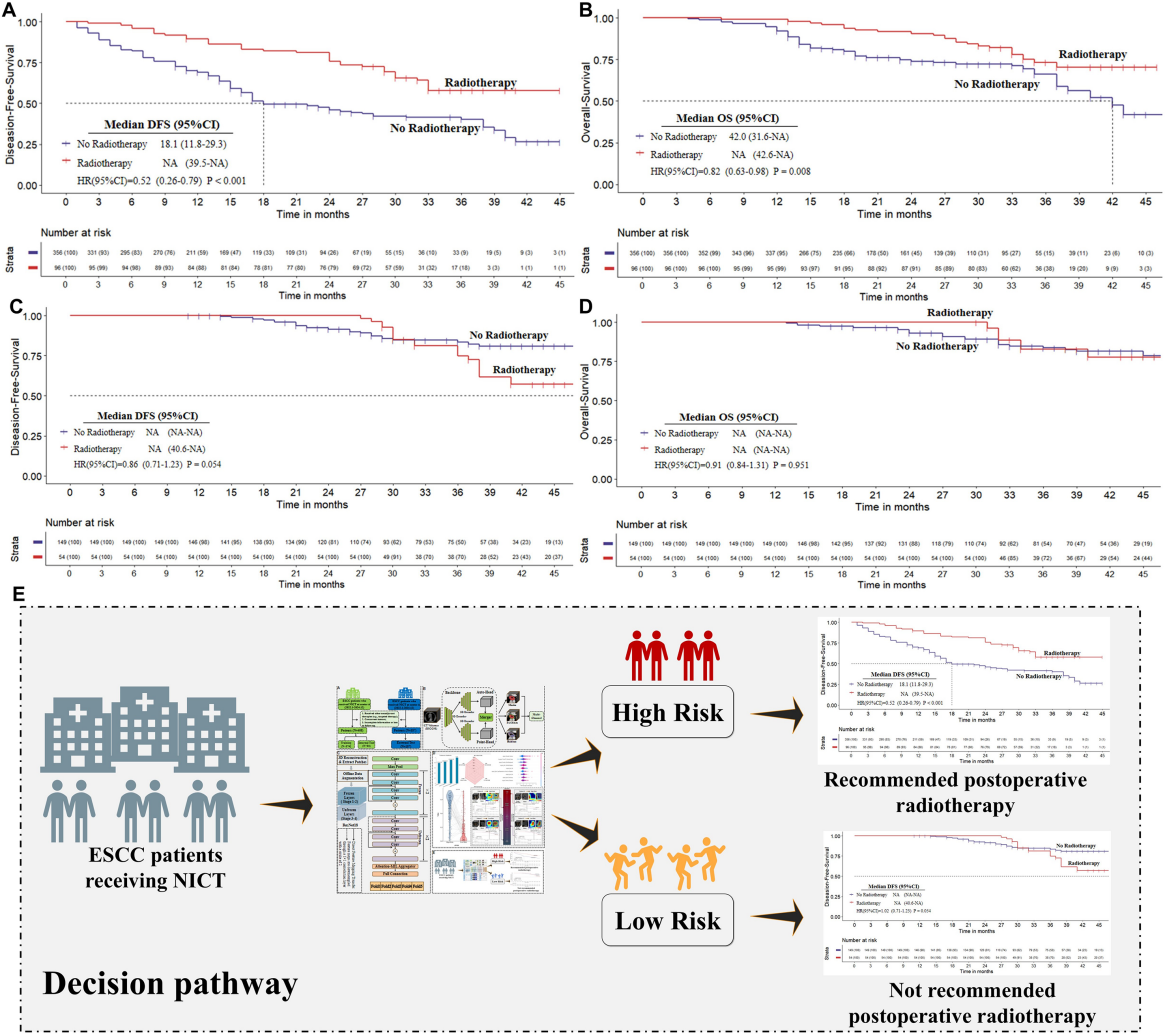
Kaplan–Meier survival analysis for whether to receive adjuvant radiotherapy and clinical decision pathway. **(A)** Kaplan–Meier survival analysis of DFS in high-risk patients according to whether to receive adjuvant radiotherapy. **(B)** Kaplan–Meier survival analysis of OS in high-risk patients according to whether to receive adjuvant radiotherapy. **(C)** Kaplan–Meier survival analysis of DFS in low-risk patients according to whether to receive adjuvant radiotherapy. **(D)** Kaplan–Meier survival analysis of OS in low-risk patients according to whether to receive adjuvant radiotherapy. **(E)** Clinical Decision Pathway. The deep learning model based on ResNet18 established in this study categorizes patients into low-risk and high-risk groups. Patients in the high-risk group are considered to have poorer prognosis following NICT and may benefit from adjuvant radiotherapy. Therefore, this study recommends that patients identified as high-risk by this model undergo adjuvant radiotherapy. Patients in the low-risk group are considered to have a favorable prognosis following NICT and are unable to benefit from adjuvant radiotherapy. Therefore, this study recommends that patients identified as low-risk by this model should carefully consider whether to undergo adjuvant radiotherapy to avoid overtreatment.

Among the 203 patients assessed as low risk by the DL model, 54 (26.60%) received radiotherapy, while 149 (73.40%) did not. Analysis of DFS revealed no significant benefit for patients receiving radiotherapy compared to those who did not (HR: 0.86 [95% CI: 0.71–1.23], P = 0.054). Similar results were observed in the OS analysis (HR: 0.91 [95% CI: 0.84–1.31], P = 0.951) (**Figures 5C, D**). The clinical decision pathway established based on this study is detailed in **Figure 6**.

## 4 Discussion

NICT is the standard treatment regimen for patients with advanced ESCC, yet most patients remain at risk of disease progression or recurrence. Accurately predicting the prognosis of patients who have received NICT and identifying those who may benefit from postoperative adjuvant radiotherapy is crucial. This approach not only improves patient outcomes but also reduces overtreatment. Therefore, this study first achieved automated tumor segmentation in ESCC patients by combining automatic segmentation with point-prompt segmentation technology, establishing a DL model for predicting prognosis. The research also found that patients identified as high-risk by the model could benefit from postoperative adjuvant radiotherapy.

Treatment strategies for locally advanced ESCC remain under investigation. Neoadjuvant combined therapy is currently the most widely adopted treatment approach. Although NCRT significantly improves pCR and 10-year OS rates, it carries a high incidence of adverse events and may lead to anastomotic leakage formation (8). Based on the Phase III ESCORT-NEO/NCCES01 clinical trial conducted by Li et al., NICT has become the standard treatment regimen for ESCC (10). The CROSS and NEOCRTEC5010 studies demonstrated that the 5-year recurrence rate in patients following NCRT reached as high as 40% (6, 7). However, adjuvant nivolumab therapy significantly improved DFS in patients with non-pCR after NCRT, indicating that postoperative adjuvant treatment is crucial for improving patient prognosis (22). Previous studies have extensively examined the efficacy of adjuvant radiotherapy in patients with ESCC who received surgery alone. Currently, adjuvant radiotherapy is primarily recommended for high-risk postoperative patients, such as those with R1/R2 resection or pN+ status (26, 27). The study by Yu et al. included patients with N+ or stage III ESCC who underwent R0 resection followed by postoperative adjuvant radiotherapy or chemoradiotherapy. Results demonstrated that compared with surgery alone, this treatment regimen significantly improved 3-year and 5-year survival rates and prolonged median survival (27). These findings indicate that adjuvant radiotherapy serves as a crucial supplementary treatment for high-risk postoperative patients. Similarly, Zeng et al. confirmed that postoperative radiotherapy significantly improved survival benefits and disease control rates in pN+ and stage III patients (28). Collectively, these discoveries suggest that adjuvant radiotherapy enhances the efficacy of local therapy and improves prognosis in patients with high-risk recurrence factors.

Previous studies have explored DL models based on CT radiomics to predict treatment efficacy (14, 29, 30). Beyond treatment-response prediction, radiomics and DL-based prognostic models have been increasingly applied to direct survival-risk stratification across tumor types. In glioma and glioblastoma, DTI/MRI-derived DL and radiomic phenotypes have been shown to stratify prognostic risk and to reflect distinct molecular pathway or transcriptomic activities (31, 32). Multi-task learning frameworks have further enabled concurrent survival prediction and semi-supervised segmentation from brain MRI (33), whereas DWI-based radiomics nomograms have been developed for preoperative prediction of PFS in muscle-invasive bladder cancer (34). In digital pathology, hybrid-graph attention networks using whole-slide pathological images and multimodal attention networks integrating whole-slide images with transcriptomic data have also demonstrated promise for survival analysis (35, 36). Despite these advances, many models remain difficult to implement in busy clinical settings, particularly when they depend on manual segmentation or complex multimodal data acquisition. With the rapid advancement of AI, replacing manual segmentation with automated AI segmentation will significantly enhance clinical applicability. In this study, when trained using the VISTA3D segmentation model, a C-index of 0.85 was achieved on the external validation cohort. The Dice similarity coefficient reached 0.87 ± 0.18, indicating that models based on automated segmentation can effectively replace manual segmentation tasks.

More importantly, the model not only effectively distinguishes patients with favorable from those with poor prognosis, but its generated DL risk score also serves as an independent predictor of DFS. Through Grad-CAM and SHAP analysis, we further revealed the decision basis of the model: predictions for high-risk patients primarily relied on imaging features of tumor perivascular invasion, while predictions for low-risk patients were more heavily based on patterns of tumor internal heterogeneity. This finding not only enhances the model’s interpretability and clinical credibility but also provides a novel imaging perspective for understanding the biological behavior and treatment response of ESCC.

Another significant contribution of this study lies in its first-ever identification of patient groups benefiting from postoperative adjuvant radiotherapy based on DL risk stratification. Among high-risk patients, postoperative adjuvant radiotherapy significantly improved DFS and OS; however, in low-risk patients, adjuvant radiotherapy did not provide significant survival benefits. This finding has important clinical implications: For patients with locally advanced ESCC receiving NICT, postoperative adjuvant radiotherapy should not be routinely recommended but should be based on individualized risk assessment. The clinical decision pathway proposed in this study provides a concrete implementation for this precision treatment concept. Specifically, it identifies high-risk patients through this model and recommends postoperative adjuvant radiotherapy for them. This approach may enhance treatment efficiency while preventing low-risk patients from enduring unnecessary radiotherapy-related toxicities and side effects.

We acknowledge that the risk stratification cutoff (0.53) used in this study was determined using the entire cohort and has not been independently validated in an external dataset. However, this cutoff was primarily employed as an exploratory tool to enable within-cohort subgroup analysis of adjuvant radiotherapy benefit. Deriving the cutoff exclusively from the training cohort would have substantially reduced statistical power due to the limited number of radiotherapy-treated patients in this subset, potentially leading to an unreliable threshold. The validity of the current cutoff is empirically supported by the significant prognostic differences observed between the low-risk and high-risk groups regarding adjuvant treatment outcomes: high-risk patients derived significant survival benefits from adjuvant radiotherapy (DFS HR: 0.52, P < 0.001; OS HR: 0.82, P = 0.008), while low-risk patients showed no such benefit (DFS HR: 0.86, P = 0.054; OS HR: 0.91, P = 0.951). These divergent outcomes confirm that the 0.53 cutoff effectively discriminates between patients with differential radiotherapy benefit.

Although this study validated the model’s effectiveness, several limitations remain. First, the retrospective design of this study may introduce selection bias. Second, although patients from two centers were included, the sample size remains limited, particularly in the subgroup receiving postoperative radiotherapy, which may affect statistical power. Third, this study relies solely on pre-treatment CT imaging for prediction, without integrating dynamic imaging changes during treatment, blood biomarkers, or genomic information. Future research may consider developing multimodal fusion models to further enhance predictive performance. Fourth, this model has not yet been validated in a prospective clinical setting, and its real-world clinical utility and actual impact on treatment decisions require further evaluation.

## 5 Conclusion

The DL model developed in this study, based on automated segmentation combined with point-prompt segmentation, can replace prediction models relying on manual segmentation. This model effectively assists clinicians in predicting the prognosis of locally advanced ESCC patients undergoing NICT, validating the feasibility of a two-stage automated prediction framework integrating “image segmentation-prognosis prediction”. Additionally, patients identified as high-risk by the model can significantly benefit from adjuvant radiotherapy. This approach supports clinical decision-making for ESCC patients, helps avoid overtreatment, and advances the progress toward personalized and precision cancer care.

## Data Availability

The raw data supporting the conclusions of this article will be made available by the authors, without undue reservation.
