# Supplementary material for: Deep learning models based on CT predict prognosis in patients with locally advanced esophageal squamous cell carcinoma who received neoadjuvant immunotherapy and chemotherapy
Source: Front Immunol. 2026 Aug 4;17:1900089. doi: 10.3389/fimmu.2026.1900089 (PMC13481367; doi:10.3389/fimmu.2026.1900089)
Supplement: Supplementary file 1 [file DataSheet1.docx]

Appendix 1

**Neoadjuvant treatment regimens**

Prior to initiating treatment, all patients underwent multidisciplinary consultations to assess their condition and select an appropriate therapeutic regimen. Both before and after neoadjuvant therapy, computed tomography (CT) or positron emission tomography-computed tomography (PET-CT) scans were performed to evaluate tumour changes. The primary preoperative chemotherapy regimens for all enrolled patients comprised the intravenous administration of platinum-based agents combined with fluorouracil (PF regimen), or platinum-based agents combined with paclitaxel or albumin-bound paclitaxel (TP regimen). The PF regimen included a platinum-based agent (nedaplatin 75 mg/m², carboplatin with an area under the curve of 5, or cisplatin 25 mg/m²), administered on days 1–3, and either fluorouracil 50 mg/m² on days 1–5 or S-1 60 mg/m²/day, administered orally twice daily on days 1–14. The TP regimen comprises docetaxel (75 mg/m²), paclitaxel (135–175 mg/m²) or albumin-bound paclitaxel (260 mg/m²). The preoperative immunotherapy regimen involves the intravenous administration of a PD-1/PD-L1 inhibitor (toripalimab 240 mg, sintilimab 200 mg, camrelizumab 200 mg, pembrolizumab 200 mg, tislelizumab 200 mg, durvalumab 1,500 mg or atezolizumab 1,200 mg). Both chemotherapy and immunotherapy involved a 3-week treatment cycle. All patients received one to three cycles of NICT preoperatively, with a median of two cycles.

**Surgical treatment**

All patients enrolled in this study were clinically assessed as suitable candidates for radical esophagectomy. Under general anaesthesia, esophagectomy and mediastinal lymph node dissection were performed four to eight weeks after the final course of neoadjuvant therapy was completed. The surgery was performed via open thoracotomy or thoracoabdominal laparoscopy, using either a two- or three-port approach. Bilateral lymph node dissection was the standard procedure. Patients with suspected cervical lymphadenopathy underwent standard trilateral lymph node dissection.

**Adjuvant treatment regimens**

All enrolled patients received postoperative adjuvant systemic therapy, with the postoperative adjuvant systemic therapy regimen identical to the preoperative chemotherapy and immunotherapy regimen. For postoperative adjuvant radiotherapy, all patients underwent positioning under large-aperture CT, with CT positioning images transferred to the Varian Eclipse planning system. Positioning for each patient was jointly performed by a radiation oncologist and a medical physicist. The radiation dose was administered at 45.0–50.4 Gy (in fractions of 1.8–2.0 Gy).
