# Supplementary material for: Deep learning models based on CT predict prognosis in patients with locally advanced esophageal squamous cell carcinoma who received neoadjuvant immunotherapy and chemotherapy
Source: Front Immunol. 2026 Aug 4;17:1900089. doi: 10.3389/fimmu.2026.1900089 (PMC13481367; doi:10.3389/fimmu.2026.1900089)
Supplement: Supplementary file 2 [file DataSheet2.docx]

Appendix 2

**VISTA3D Segmentation Deep Learning Model**

This paper employs VISTA3D—a unified segmentation foundation model for 3D medical imaging—based on the SegResNet backbone and sliding-window inference. By integrating 3D super-voxel distillation (SAM) knowledge and a four-stage training approach, it achieves automatic segmentation of 127 structural classes, 3D interactive refinement, and zero-shot segmentation. Its inference speed is 3 to 4 times faster than TotalSegmentator. It demonstrates outstanding performance in few-shot fine-tuning, providing a comprehensive workflow solution for clinical 3D image segmentation. The VISTA3D model was fine-tuned on data from Center A, while data from Centers B and C continued to serve as external test sets for the object segmentation task. The preprocessing and training workflow was designed to enhance data validity and optimize model performance: First, key regions were preserved through foreground cropping. Image intensities were then normalized to the range [0, 1] from [-110, 190]. to the range [0, 1]. Simultaneously, multi-modal data augmentation techniques are introduced, including random cropping by label category, random scaling, Gaussian smoothing, intensity scaling and offset, and Gaussian noise addition, to enhance the model's generalization capability.

Model and training hyperparameters were tailored to the segmentation task requirements: The optimizer employed AdamW with weight decay, an initial learning rate of 5e-5, and a WarmupCosineSchedule learning rate scheduler. The loss function utilized DiceCELoss (excluding background, with sigmoid activation and squared prediction enabled, smooth_dr=1e-5). During training, the batch size was configured as 8 images per batch over 200 total epochs, with mixed precision training enabled. In the fine-tuning phase, pre-trained weights were loaded to ensure efficient convergence and adaptation to the segmentation task requirements on both Center A and Center C datasets.

**Reliability Analysis of Segmentation Results**

The reliability analysis in this study focuses on the results of three core segmentation methods supported by VISTA3D compared against manually annotated gold standards on an external test cohort. The specific sources are as follows: First, automatic segmentation relies on the shared SegResNet backbone and automatic branching to achieve end-to-end segmentation of target structures without additional manual intervention; Second, point-prompt segmentation (interactive segmentation) activates an interactive branch by inputting 3D positive/negative point prompts, flexibly adapting to segmentation demands for new categories not covered by the automatic branch. Third, automatic segmentation + point-prompt segmentation employs a model-specific merging algorithm to use point prompts solely for correcting false positive/false negative regions in the automatic segmentation, avoiding disruption of correctly segmented areas.

To prevent data bias from interfering with reliability analysis, all segmentation results must meet the following preprocessing consistency requirements:

1. Spatial coordinate alignment: Map all three segmentation masks and manually segmented gold standards to the coordinate system of the same CT image (based on positioning parameters in DICOM files, ensuring pixel coordinates correspond one-to-one with anatomical locations);

2. Resolution standardization: Resample all images and segmentation masks to a 1mm×1mm×1mm isotropic resolution;

3. Region cropping: Retain only segmentation masks within the largest connected region containing the tumor area.

Four widely recognized quantitative metrics in medical image segmentation were selected: Dice similarity coefficient, 95% Hausdorff distance, mean surface distance, and volume similarity. These metrics were used to calculate the consistency between the three segmentation results and the manually segmented gold standard.
