# Supplementary material for: Deep learning models based on CT predict prognosis in patients with locally advanced esophageal squamous cell carcinoma who received neoadjuvant immunotherapy and chemotherapy
Source: Front Immunol. 2026 Aug 4;17:1900089. doi: 10.3389/fimmu.2026.1900089 (PMC13481367; doi:10.3389/fimmu.2026.1900089)
Supplement: Supplementary file 3 [file DataSheet3.docx]

Appendix 3

**Network Architecture and Training Configuration**

The pydicom library was used to read DICOM slice sequences from each patient's folder. All slices were numerically sorted to accurately reconstruct anatomically continuous 3D CT volumes. This study employs ResNet18 (an 18-layer residual network pre-trained on ImageNet) as the feature extraction backbone. To balance the retention of pre-trained knowledge with adaptation to CT image features, a hierarchical freezing strategy was adopted. Specifically, the model was divided into five stages according to the ResNet18 architecture: stage 1 (conv1, stride 2), stage 2 (layer1, two basic blocks), stage 3 (layer2, two basic blocks), stage 4 (layer3, two basic blocks), and stage 5 (layer4, two basic blocks). In the final configuration, stages 1 through 3 were frozen (i.e., no gradient updates were applied to these layers), while stages 4 and 5 were unfrozen and fine-tuned during training. This configuration was empirically selected based on preliminary experiments comparing different freezing strategies (freezing 0–5 stages), with the final choice yielding the best validation C-index.

Training was performed using the Adam optimizer with an initial learning rate of 1 × 10⁻⁴, which was reduced by a factor of 0.5 upon plateau in validation loss (patience: 5 epochs). A batch size of 16 was used, and the model was trained for a maximum of 100 epochs with early stopping based on validation C-index (patience: 15 epochs). We also employed L2 regularization (weight decay: 1 × 10⁻⁵) to mitigate overfitting. All training procedures were implemented using PyTorch (version 2.0.1).

We implemented an aggregator based on AttentionMIL to aggregate instance-level features into patient-level representations. For each patient, the extracted slice-level features (dimension: 512, from the ResNet18 global average pooling layer) were stacked to form a bag of instances. The attention-based aggregator consists of a two-layer Multi-Layer Perceptron (MLP) with hidden dimension 256 and output dimension 1, followed by a Softmax activation function to compute instance-specific attention weights. These weights were used to compute a weighted sum of the instance features, producing a patient-level feature vector of dimension 512. This patient-level representation was then passed through a final linear layer (512 → 1) with a Sigmoid activation to produce the DL risk score. Dropout (rate: 0.5) was applied after the MLP in the attention module and after the final linear layer for regularization. The attention mechanism enables the model to autonomously learn, in a data-driven manner, which patch regions (e.g., peripheral areas containing peritumoral context) contribute most significantly to predicting high risk. This generates a more information-dense and discriminative patient-level representation. In the exploratory experiments of this study, a key finding was that complex prediction heads were highly prone to overfitting on our dataset. Consequently, a simplified MLP with strong regularization was adopted as the prediction head. Simplifying the prediction head significantly reduced the number of parameters in the stage from feature aggregation to the final risk score, compelling the model to learn more robust and generalizable features at the backbone and aggregator stages.

**Model Evaluation and Hyperparameter Summary**

This study employs the Cox Proportional Hazards Loss Function (CoxPHLoss) to optimize the model. This loss function is a biased log-likelihood that does not directly predict survival time but instead optimizes the ranking of patient risks. Standard regression losses (such as MSE) cannot handle this type of incomplete data. CoxPH Loss is the gold standard for survival analysis, as it correctly utilizes information from both "event occurrence" (death=1) and "censoring" (death=0) patient categories to maximize the ranking consistency of the model (high risk and short survival). No additional weighting was applied to the loss function, as all patients were assigned equal importance during training. The loss function was implemented using the implementation available in the PyTorch Survival Analysis library (torchsurv, version 0.1.0).

During the training phase, this study incorporated an online data augmentation strategy. This approach involves applying random online transformations (such as horizontal flipping and minor translations) to each loaded patch—including offline-augmented copies—during training, thereby further mitigating overfitting in the small-sample dataset. This study further applies the Mixup strategy (alpha = 0.2) to generate "synthetic" features between pairs of samples within each batch. By creating a continuous, smooth distribution of samples in the feature space, this approach significantly enhances the model's generalization capability.

This study employed 5-fold cross-validation to evaluate model performance. During data fold partitioning, stratification was performed based on the "death" event indicator to ensure that the ratio of events to censored cases in each fold remained consistent with the overall dataset. The primary evaluation metric was Harrell's C-index, which quantifies the concordance between the model's predicted risk rankings and the actual observed survival time rankings.

To deeply understand the basis for model decisions and enhance its clinical credibility, this study employs a combined approach of Gradient-Weighted Class Activation Maps (Grad-CAM) and SHapley Additive Explanations (SHAP). This approach explains the model's predictive behavior from two complementary dimensions: spatial regions within the image and feature importance.

For Grad-CAM visualization, we selected the final convolutional layer (layer4.1.conv2) of the ResNet18 backbone as the target layer, as it captures the highest-level spatial feature maps before global pooling. For each input CT slice, we computed the gradient of the predicted risk score with respect to the feature maps of the target layer. The gradients were globally averaged over each feature map to obtain neuron importance weights, which were then used to generate a weighted combination of the feature maps. The resulting heatmap was passed through a ReLU activation function to retain only positive contributions and upsampled to the original input image resolution (512 × 512 pixels) using bilinear interpolation. The heatmap was then overlaid onto the original CT image with a transparency ratio of 0.5 (jet colormap, opacity range: 0.3–0.8) to generate the final visualization. To ensure representative and statistically meaningful results, we generated Grad-CAM heatmaps for 200 randomly selected cases from the entire cohort (100 high-risk and 100 low-risk patients, stratified by the DL score cutoff). The patterns were qualitatively summarized by two independent reviewers to identify consistent spatial attention characteristics within each risk group.

For SHAP analysis, we extracted 1,518 radiomic features from the segmented tumor and peritumoral regions, including first-order statistics, shape features, and texture features (Gray-Level Co-occurrence Matrix, Gray-Level Run Length Matrix, Gray-Level Size Zone Matrix, Gray-Level Dependence Matrix, and Neighboring Gray Tone Difference Matrix). Feature screening was performed prior to SHAP computation using the following criteria: (i) features with zero variance or near-zero variance (variance < 0.01) across the training cohort were excluded; (ii) features with an interquartile range of zero were removed; and (iii) among pairwise features with Spearman correlation coefficient > 0.90, only the feature with the higher correlation with DFS (based on univariate Cox regression) was retained. This procedure yielded 186 features for SHAP analysis. SHAP values were computed using the KernelExplainer from the SHAP library (version 0.41.0), with a background dataset of 100 randomly sampled training instances to approximate the expected model output. For each feature, we calculated the mean absolute SHAP value across all test samples as a global measure of feature importance. The SHAP summary plot was generated displaying the top 20 features ranked by mean absolute SHAP value. All SHAP analyses were performed on the external validation cohort to avoid overfitting and ensure generalizable interpretation. By combining Grad-CAM spatial localization with SHAP feature attribution, we provide a multi-dimensional interpretable framework that links imaging phenotypes to biological characteristics of tumor invasion, microenvironmental heterogeneity, and immunochemotherapeutic response.
