# Supplementary material for: Deep learning models based on CT predict prognosis in patients with locally advanced esophageal squamous cell carcinoma who received neoadjuvant immunotherapy and chemotherapy
Source: Front Immunol. 2026 Aug 4;17:1900089. doi: 10.3389/fimmu.2026.1900089 (PMC13481367; doi:10.3389/fimmu.2026.1900089)
Supplement: Supplementary file 4 [file Supplementaryfile1.docx]

**Supplementary Table S1. Five-fold stratified cross-validation performance of the DL model on the training cohort**

| **Fold** | **C-index** | **ACC** | **SEN** | **SPE** | **NPV** | **PPV** |
| --- | --- | --- | --- | --- | --- | --- |
| 1 | 0.82 | 0.76 | 0.68 | 0.77 | 0.81 | 0.72 |
| 2 | 0.86 | 0.80 | 0.73 | 0.81 | 0.84 | 0.76 |
| 3 | 0.88 | 0.83 | 0.77 | 0.83 | 0.87 | 0.79 |
| 4 | 0.84 | 0.78 | 0.71 | 0.79 | 0.82 | 0.74 |
| 5 | 0.85 | 0.78 | 0.71 | 0.80 | 0.82 | 0.75 |
| Mean ± SD | 0.85 ± 0.03 | 0.79 ± 0.04 | 0.72 ± 0.05 | 0.80 ± 0.04 | 0.83 ± 0.03 | 0.75 ± 0.03 |
